# Putative glymphatic dysfunction links extracellular fluid dysregulation to white matter degeneration and clinical impairment in amyotrophic lateral sclerosis

**DOI:** 10.1186/s12916-026-04948-z

**Published:** 2026-05-27

**Authors:** Xin Jin, Yan Fu, Ting Qiu, Jianyu Li, Huixiong Zhang, Yifan Chen, Kewei Chen, Yuanchao Zhang, Junling Wang, Xiaoping Yi, Lena Palaniyappan, B. Blair Braden

**Affiliations:** 1 The Clinical Hospital of Chengdu Brain Science Institute, MOE Key Lab for Neuroinformation, University of Electronic Science and Technology of China, Chengdu, Sichuan P. R. China; 2 School of Life Science and Technology, University of Electronic Science and Technology of China, Chengdu, 610054 Sichuan P. R. China; 3 Department of Radiology, Tongji Hospital, Tongji Medical College, Huazhong University of Science and Technology, Wuhan, P. R. China; 4 Douglas Mental Health University Institute, McGill University, Montreal, QC Canada; 5 College of Health Solutions, Arizona State University, Phoenix, AZ 85287 USA; 6 Department of Neurology, Xiangya Hospital, Central South University, Jiangxi. (National Regional Center for Neurological Diseases), Nanchang, 330038 Jiangxi P. R. China; 7 National Clinical Research Center for Geriatric Diseases(Xiangya Hospital of Central South University), Changsha, 410008 Hunan P. R. China; 8 Key Laboratory of Hunan Province for Neurodegenerative Disorders, Central South University, Changsha, 410008 Hunan P. R. China; 9 Center for Medical Genetics, School of Life Sciences, Central South University, Changsha, 410008 Hunan P. R. China; 10 Engineering Research Center of Hunan Province for Cognitive Impairment Disorders, Central South University, Changsha, 410008 Hunan P. R. China; 11 Hunan International Scientific and Technological Cooperation Base of Neurodegenerative and Neurogenetic Diseases, Changsha, 410008 Hunan P. R. China; 12 Department of Nuclear Medicine and Department of Radiology, Chongqing University Three Gorges Hospital, Chongqing University, Chongqing, 404000 P. R. China; 13 Clinical Research Center (CRC), Medical Pathology Center (MPC), Cancer Early Detection and Treatment Center (CEDTC) and Translational Medicine Research Center (TMRC), Chongqing University Three Gorges Hospital, Chongqing University, Chongqing, 404000 P.R. China; 14 School of Medicine, Chongqing University, Chongqing, 400030 P.R. China

**Keywords:** ALS, Glymphatic system, White matter integrity, DTI-ALPS, Free-water fraction

## Abstract

**Background:**

Amyotrophic lateral sclerosis (ALS) is a fatal neurodegenerative disorder characterized by progressive motor neuron degeneration and prominent extra-motor involvement. Impaired clearance of neurotoxic proteins has led to increasing interest in the brain glymphatic system; however, its in vivo associations with brain microstructure and clinical heterogeneity remain incompletely understood.

**Methods:**

One hundred forty-six patients with ALS and 149 demographically matched healthy controls (HCs) underwent multimodal MRI and comprehensive clinical assessments. Putative glymphatic function was quantified using diffusion tensor imaging along perivascular space (DTI-ALPS). Extracellular free water fraction (FWF) and free-water-corrected fractional anisotropy (fwcFA) were derived to characterize extracellular fluid and white matter microstructure. Group differences were assessed using vertex-wise and voxel-wise analyses with correction for multiple comparisons. Associations among imaging metrics and clinical measures were evaluated using correlation and serial mediation analyses.

**Results:**

Compared with HCs, patients with ALS exhibited significantly reduced DTI-ALPS index, widespread increases in cortical FWF, bidirectional alterations in white matter FWF, and extensive reductions in fwcFA across major white matter tracts. Reduced DTI-ALPS was associated with changes in extracellular free water and white matter microstructural integrity, whereas FWF and fwcFA measures were associated with functional, cognitive, and emotional outcomes. Mediation analyses identified significant indirect associations between DTI-ALPS and both functional and cognitive measures through a pathway involving cortical FWF, white matter FWF, and fwcFA, although direct associations were not observed.

**Conclusions:**

These findings provide in vivo evidence that putative glymphatic dysfunction co-occurs with extracellular fluid alterations, white matter microstructural changes, and clinical impairment in ALS. Multi-compartment diffusion imaging may offer complementary markers for characterizing brain microstructure and its clinical relevance in ALS.

**Supplementary Information:**

The online version contains supplementary material available at 10.1186/s12916-026-04948-z.

## Background

 Amyotrophic lateral sclerosis (ALS) is a fatal and progressive neurodegenerative disorder characterized by degeneration of upper and lower motor neurons, leading to muscle weakness, paralysis, and ultimately respiratory failure [1]. Although ALS primarily affects the motor system, growing evidence indicates widespread extra-motor involvement, including cognitive and behavioral impairments that overlap with the frontotemporal dementia spectrum [2, 3]. Despite decades of research, the precise mechanisms underlying ALS remain incompletely understood, and effective disease-modifying therapies are still lacking. A pathological hallmark in over 90% of ALS cases is cytoplasmic mislocalization and aggregation of the RNA-binding protein TDP-43, which disrupts RNA metabolism and neuronal homeostasis [3, 4]. While TDP-43 pathology is central to ALS, its accumulation may also reflect impaired clearance of neurotoxic proteins [5–7], raising the possibility that dysfunction in brain fluid and waste removal systems contributes to disease progression.

Recent research has highlighted the potential involvement of the brain’s glymphatic system—a perivascular network responsible for clearing metabolic waste and maintaining interstitial fluid homeostasis [8–11]—in the pathophysiology of ALS. The glymphatic system facilitates cerebrospinal fluid (CSF) influx along periarterial spaces and interstitial solute clearance along perivenous routes, processes essential for removing neurotoxic proteins such as TDP-43 and SOD1. Evidence from transgenic ALS mouse models [12, 13] demonstrates that glymphatic transport becomes impaired early in the disease course, leading to stagnation of interstitial flow and delayed clearance of misfolded proteins. Consistent with these preclinical findings, multimodal MRI studies in ALS patients [14, 15]—including brain-CSF coupling and diffusion tensor imaging along perivascular spaces (DTI-ALPS)—have revealed reduced glymphatic activity, which correlates with motor disability, cognitive decline, and overall disease severity [16]. These observations suggest that glymphatic dysfunction may contribute to both motor and extra-motor manifestations of ALS, potentially through extracellular fluid accumulation, neuroinflammation, and progressive microstructural degeneration. Given that these imaging metrics represent indirect proxies rather than definitive measures of glymphatic function, we refer here to the observed alterations as putative glymphatic dysfunction.

Free water (FW) imaging, derived from diffusion MRI, estimates the fractional volume of extracellular (unrestricted) water and serves as a sensitive in vivo marker of neuroinflammation, extracellular edema, and microstructural tissue loss [17–20]. FW increases have been linked to cognitive and behavioral decline across aging and neurodegenerative conditions [21, 22] and correlate with molecular markers of neuroinflammation and tau pathology in Alzheimer’s disease [23–25], underscoring its pathophysiological relevance. Importantly, FW alterations appear to co-vary with impaired perivascular fluid dynamics: studies report associations among enlarged perivascular spaces, reduced DTI-ALPS indices, and elevated FW fractions, suggesting that compromised glymphatic clearance may lead to interstitial fluid accumulation detectable as increased FW [25–27]. Integrating DTI-ALPS, FW, and fractional anisotropy (FA) metrics in ALS therefore offers a unique opportunity to test whether impaired glymphatic transport is associated with extracellular fluid expansion and consequent white matter microstructural degeneration—thereby linking disrupted fluid dynamics to disease progression.

The primary aim of this study was to elucidate how putative glymphatic dysfunction, as inferred from the DTI-ALPS index, relates to extracellular FW accumulation, white matter microstructural integrity, and clinical characteristics in patients with ALS compared with healthy controls. Building on prior evidence and theoretical considerations [16, 28], we formulated three hypotheses. First, patients with ALS would exhibit significant putative glymphatic dysfunction, along with increased extracellular free water accumulation and decreased white matter microstructural integrity (lower free-water corrected FA [fwcFA]) compared with healthy controls. Second, alterations in putative glymphatic function were expected to parallel clinical manifestations, such that lower DTI-ALPS values and abnormal FW or fwcFA metrics would be associated with greater disease severity, cognitive decline, and emotional disturbance. Third, the relationship between putative glymphatic dysfunction and clinical outcomes would be mediated by FW accumulation and disrupted white matter microstructure, potentially following a pathway involving cortical FW, white matter FW, and fwcFA. This hypothesis was motivated by prior studies [25, 29] that have applied similar mediation frameworks linking DTI-ALPS, FW, and white matter integrity to cognitive outcomes in aging and neurodegenerative populations.

## Methods

### Participants

A total of 146 patients with ALS and 149 demographically matched healthy controls (HCs) were included in the present study. Specifically, patients were consecutively recruited from the Department of Neurology at Xiangya Hospital, Central South University, China. All patients were diagnosed with clinically definite, probable, or probable laboratory-supported sporadic ALS using the revised 2015 El Escorial criteria [30], confirmed independently by two experienced neurologists. All participants underwent standardized neurological and neuropsychological examinations on the day of imaging acquisition. Individuals were excluded if they had a family history of any neurological disorder; or any major systemic, psychiatric, or neurologic condition such as epilepsy, stroke, or structural brain abnormalities; or other causes of focal or diffuse brain damage, including lacunar infarctions or extensive cerebrovascular disease evident on routine MRI. Demographic and clinical data were collected at the time of screening, including age at onset, site of onset, time from onset to diagnosis, and scores on the Amyotrophic Lateral Sclerosis Functional Rating Scale-Revised (ALSFRS-R) [31], King’s College staging system [32], Mini-Mental State Examination (MMSE) [33], Patient Health Questionnaire-9 (PHQ-9) [34] and Generalized Anxiety Disorder 7-item (GAD-7) [35].

### MRI acquisition

All MRI data were acquired on a 3.0-T Siemens Prisma scanner at Xiangya Hospital. T1-weighted images were obtained using a three-dimensional magnetization-prepared rapid gradient-echo sequence with 1 mm isotropic voxels (matrix = 256 × 256, slice thickness = 1 mm, repetition time [TR] = 2300 ms, echo time [TE] = 2.98 ms, inversion time [TI] = 900 ms, flip angle = 9°, field of view [FOV] = 256 × 256 mm², no gap). Diffusion-weighted images were acquired using a spin-echo echo-planar imaging sequence, including b = 0 s/mm² images, 64 diffusion-weighted directions at b = 1000 s/mm², and 64 directions at b = 2000 s/mm². Imaging parameters were: TR = 4300 ms, TE = 75 ms, matrix = 132 × 132, FOV = 264 × 264 mm², slice thickness = 2 mm, and 70 axial slices without a slice gap. Participants were instructed to keep their eyes closed, remain still, and minimize head motion during scanning.

### MRI data preprocessing

T1-weighted images for all participants were processed using the standard recon-all pipeline in FreeSurfer (v7.4.1; http://surfer.nmr.mgh.harvard.edu/). Briefly, the pipeline included intensity normalization, skull stripping, and gray/white matter segmentation, followed by topology-corrected reconstruction of the white and pial surfaces and automated anatomical parcellation. All reconstructions underwent visual quality control, and any issues (e.g., incomplete skull stripping, tissue misclassification, or surface defects) were corrected manually before re-running the relevant recon-all steps.

Diffusion-weighted images were preprocessed using the publicly available DTI-ALPS pipeline (https://github.com/gbarisano/alps/) [36, 37]. Briefly, denoising and Gibbs-ringing removal were performed using MRtrix3, followed by eddy current and head-motion correction in FSL. Diffusion tensor model was then fitted for each participant to generate FA and diffusivity maps along the x-, y-, and z-axes. The FA maps were co-registered to the JHU-ICBM-FA template using linear and nonlinear registration tools in FSL, and all resulting diffusion-derived maps were visually inspected to ensure the absence of artifacts or processing errors. Based on the JHU-ICBM-DTI-81 white-matter atlas, 5-mm spherical regions of interest (ROIs) were automatically placed in the bilateral superior corona radiata (SCR) and superior longitudinal fasciculus (SLF) at the level of the lateral ventricle body. Diffusivity values (Dxx, Dyy, Dzz) were extracted from these ROIs to calculate the DTI-ALPS index. The DTI-ALPS index is defined as the mean of the bilateral indices and is calculated as the ratio of the mean x-axis diffusivity in the projection and association fibers (Dxx_proj and Dxx_assoc) to the mean diffusivity perpendicular to both the perivascular space and local white-matter fiber directions, represented by the y-axis diffusivity in projection fibers (Dyy_proj) and the z-axis diffusivity in association fibers (Dzz_assoc).

### FW mapping

FW estimation was performed on eddy current-corrected diffusion-weighted images using the free-water elimination model in DIPY (https://docs.dipy.org/stable/examples_built/reconstruction/reconst_fwdti.html), which separates tissue-specific diffusion from extracellular free-water contributions. This approach yields fwcFA and the FW fraction (FWF), providing measures of microstructural integrity and extracellular FW content across both white and gray matter. By accounting for the contribution of extracellular FW, fwcFA reduces biases in conventional FA measures caused by partial volume effects from CSF, particularly in regions adjacent to the ventricles and brain parenchyma, while FWF estimates the proportion of extracellular free water in these tissues.

### MRI quality control

All diffusion-weighted imaging data underwent comprehensive quality control procedures. Intermediate outputs from each automated preprocessing step were visually inspected to ensure data integrity, accurate preprocessing, proper spatial alignment, and the absence of significant motion-related artifacts. No participants were excluded based on these quality assessments.

### Statistical analyses

Differences in sex distribution between the two groups were evaluated using the chi-square (χ²) test. Continuous variables (age, MMSE, PHQ-9, GAD-7 scores, and the DTI-ALPS index) were first assessed for normality using the Shapiro–Wilk test. Variables satisfying normality assumptions were analyzed using two-sample t-tests, whereas non-normally distributed variables were analyzed using non-parametric Mann–Whitney U tests, as appropriate. All analyses were performed using SciPy (https://scipy.org/), with statistical significance defined as *p* < 0.05.

Vertex-wise analysis of cortical FWF was conducted using SurfStat (https://www.math.mcgill.ca/keith/surfstat/) in MATLAB. Specifically, each participant’s FWF image was first registered to the corresponding preprocessed T1-weighted structural image using the bbregister pipeline. The volumetric FWF values were then projected onto the individual cortical surface and subsequently mapped to the standard fsaverage surface for vertex-wise analysis. At each vertex, a linear model was constructed with group as the independent variable. Multiple comparisons were corrected using Random Field Theory, applying a cluster-forming threshold of *p* < 0.001. The mean FWF within all significant clusters was subsequently extracted for each participant and used in further analyses.

Voxel-wise analysis of white matter fwcFA and FWF was performed using the Tract-Based Spatial Statistics (TBSS) pipeline in FSL. All fwcFA maps were first registered to the TBSS-provided FMRIB58-FA template using ANTs (https://github.com/ANTsX/ANTs) through a three-step procedure (rigid, affine, and SyN nonlinear registration), and all resulting diffusion maps were visually inspected for artifacts or processing errors. A mean fwcFA image was skeletonized (thresholded at fwcFA > 0.2) to represent core white matter tracts, and individual fwcFA data were projected onto this skeleton. FWF maps were processed using the same transformations to maintain spatial correspondence. Group comparisons were conducted using non-parametric permutation testing (randomise, 5000 permutations), with multiple comparisons corrected via threshold-free cluster enhancement (TFCE). White matter tracts were identified based on the JHU-ICBM [38, 39] white matter tractography atlas, specifically using the tracts-maxprob-thr25 template. For subsequent analyses, mean fwcFA values were extracted across all significant white matter regions, and for FWF, mean values were separately extracted from all significant white matter regions showing increased or decreased FWF relative to controls.

Within the patient group, associations among imaging metrics (DTI-ALPS index, mean fwcFA, and mean FWF values extracted from affected regions/tracts) and clinical measures (ALSFRS-R, MMSE, PHQ-9, and GAD-7) were assessed using Pearson’s correlation for normally distributed variables and Spearman’s rank correlation for non-normally distributed variables, as appropriate. These results were further corrected for multiple comparisons using the false discovery rate (FDR) method. Building on the observed correlations, two serial multiple mediation analyses were conducted in SPSS (PROCESS macro, Version 4.1, www.afhayes.com; Model 6) to assess whether FWF_GM_ (mean FWF increase in cortical gray matter), FWF_WM_ (mean FWF increase in white matter), and the mean reduction in white matter fwcFA mediated the effects of the DTI-ALPS index on ALSFRS-R and MMSE. Specifically, the DTI-ALPS index was specified as the independent variable (X), FWF_GM_, FWF_WM_, and fwcFA as the first to third mediators (M1–M3), respectively, and ALSFRS-R or MMSE as the outcome variable (Y), corresponding to the pathway: DTI-ALPS → FWF_GM_ → FWF_WM_→ fwcFA → ALSFRS-R/MMSE. However, this represents one of several biologically plausible models, and alternative explanations—such as parallel processes, reverse associations, or shared upstream mechanisms—cannot be excluded. Indirect, direct, and total effects were estimated using nonparametric bootstrapping with 5,000 resamples, and 95% percentile bootstrap confidence intervals were calculated. Effects were considered statistically significant when the confidence intervals (CIs) did not include zero.

## Results

### Demographic and clinical profiles

There were no significant differences in age (Z = 0.785, *p* = 0.432, Cliff’s δ = 0.053) or sex distribution (χ² = 0.007, *p* = 0.935, Cramér’s V = 0.005) between patients with ALS and HCs (Table 1). Compared with HCs, patients with ALS showed significantly lower MMSE scores (Z=-3.956, *p* < 0.001, Cliff’s δ=-0.263), as well as significantly higher PHQ-9 (Z = 7.461, *p* < 0.001, Cliff’s δ = 0.486) and GAD-7 (Z = 5.327, *p* < 0.001, Cliff’s δ = 0.351) scores, indicating greater cognitive impairment and more severe emotional disturbances.

**Table 1:** Demographic and clinical features of patients with ALS and HC

	Patients with ALS (*n* = 146)	HCs (*n* = 149)	Statistic	*p*-value	Effect size
Age, y	55.54 ± 10.26	54.72 ± 9.49	Z = 0.785	*p* = 0.432	δ = 0.053
Male/Female	83/63	84/65	χ²=0.007	*p* = 0.935	V = 0.005
ALSFRS-R	40.53 ± 5.21				
ΔFS	0.73 ± 0.78				
MMSE	25.95 ± 4.18	27.93 ± 1.51	Z=-3.956	*p* < 0.001	δ=-0.263
PHQ-9	4.77 ± 5.74	0.95 ± 1.39	Z = 7.461	*p* < 0.001	δ = 0.486
GAD-7	4.79 ± 5.91	1.40 ± 1.64	Z = 5.327	*p* < 0.001	δ = 0.351

Note: Data represent mean ± standard deviation. Sex differences were assessed using the χ² test, while continuous variables were analysed using the Mann–Whitney U test. Effect sizes were calculated using Cliff’s delta and Cramér’s V. ALSFRS-R Amyotrophic Lateral Sclerosis Functional Rating Scale-Revised, ΔFS progression rate of ALS, MMSE Mini-Mental State Examination, PHQ-9 Patient Health Questionnaire-9, GAD-7 General Anxiety Disorder-7

### Decreased DTI-ALPS in ALS

The DTI-ALPS index was significantly lower (t = 3.954, *p* < 0.001, Cohen’s *d* = 0.460) in patients with ALS (1.41 ± 0.14) compared to HCs (1.48 ± 0.16), suggesting putative glymphatic dysfunction in patients with ALS.

### Alterations in FWF in ALS

**Fig. 1:**
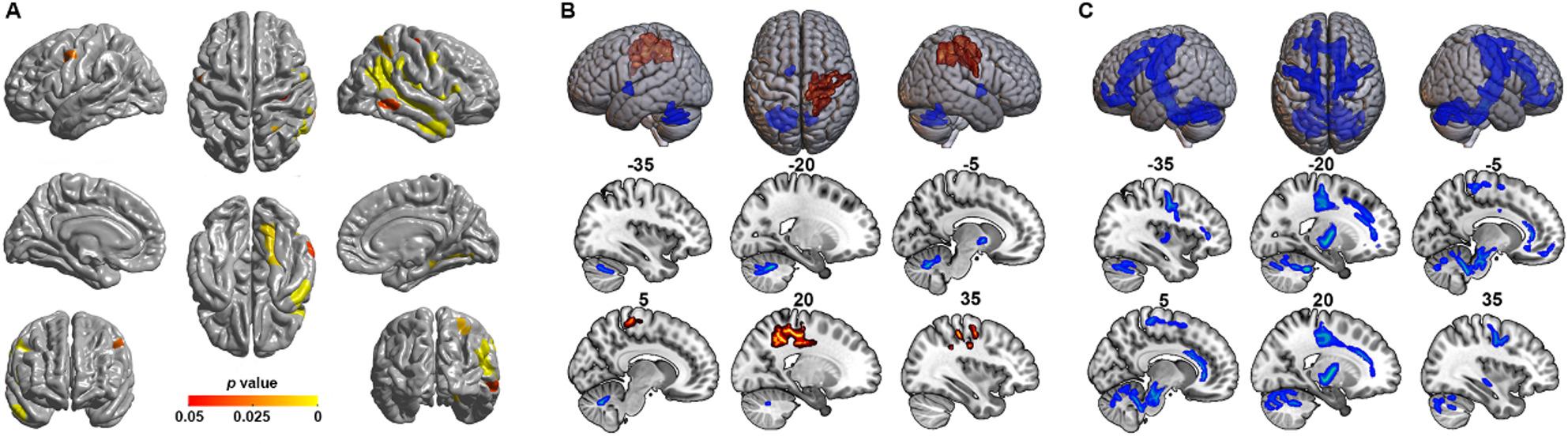
Between-group differences in FWF and fwcFA. **A** Cortical regions showing significantly higher free-water fraction (FWF) in patients with ALS compared with healthy controls. Results were corrected for multiple comparisons using random field theory (RFT), with significance set at RFT-corrected *p* < 0.05. **B** White matter regions showing significant differences in FWF between patients with ALS and healthy controls. Warm colors indicate higher FWF in patients with ALS relative to healthy controls, whereas cold colors indicate lower FWF in patients with ALS. Results were corrected for multiple comparisons using threshold-free cluster enhancement (TFCE), with significance set at TFCE-corrected *p* < 0.05. **C** White matter regions showing significantly lower fwcFA in patients with ALS compared with healthy controls. Results were corrected for multiple comparisons using TFCE, with significance set at TFCE-corrected *p* < 0.05. FWF, free-water fraction; fwcFA, FW-corrected fractional anisotropy

Compared to HCs, patients with ALS showed significantly increased FWF in multiple cortical regions (Fig. 1A), including the precentral gyrus, paracentral lobule, orbitofrontal cortex, and the superior, middle, and inferior frontal gyri, as well as the superior parietal lobule, angular gyrus, and parahippocampal gyrus. The effect size map for the contrast is provided in Additional file 1: Fig. S1.

In the white matter, patients with ALS exhibited significant bidirectional alterations in FWF (Fig. 1B). Specifically, increased FWF was observed in the right corticospinal tract (CST) and right SLF, whereas decreased FWF was detected in the left anterior thalamic radiation and cerebellum. The effect size map for the contrast is provided in Additional file 1: Fig. S2.

### Decreased fwcFA in ALS

Compared to HCs, patients with ALS exhibited widespread reductions in white matter fwcFA (Fig. 1C), primarily involving the anterior thalamic radiation, corticospinal tract, cingulum, forceps minor, inferior fronto-occipital fasciculus, SLF, uncinate fasciculus, and bilateral cerebellum. The effect size map for the contrast is provided in Additional file 1: Fig. S3.

### Relationships between imaging and clinical data

In patients with ALS, a lower DTI-ALPS index was significantly associated with a greater ΔFS (*r* = -0.1742, uncorrected *p* = 0.0355).

**Fig. 2:**
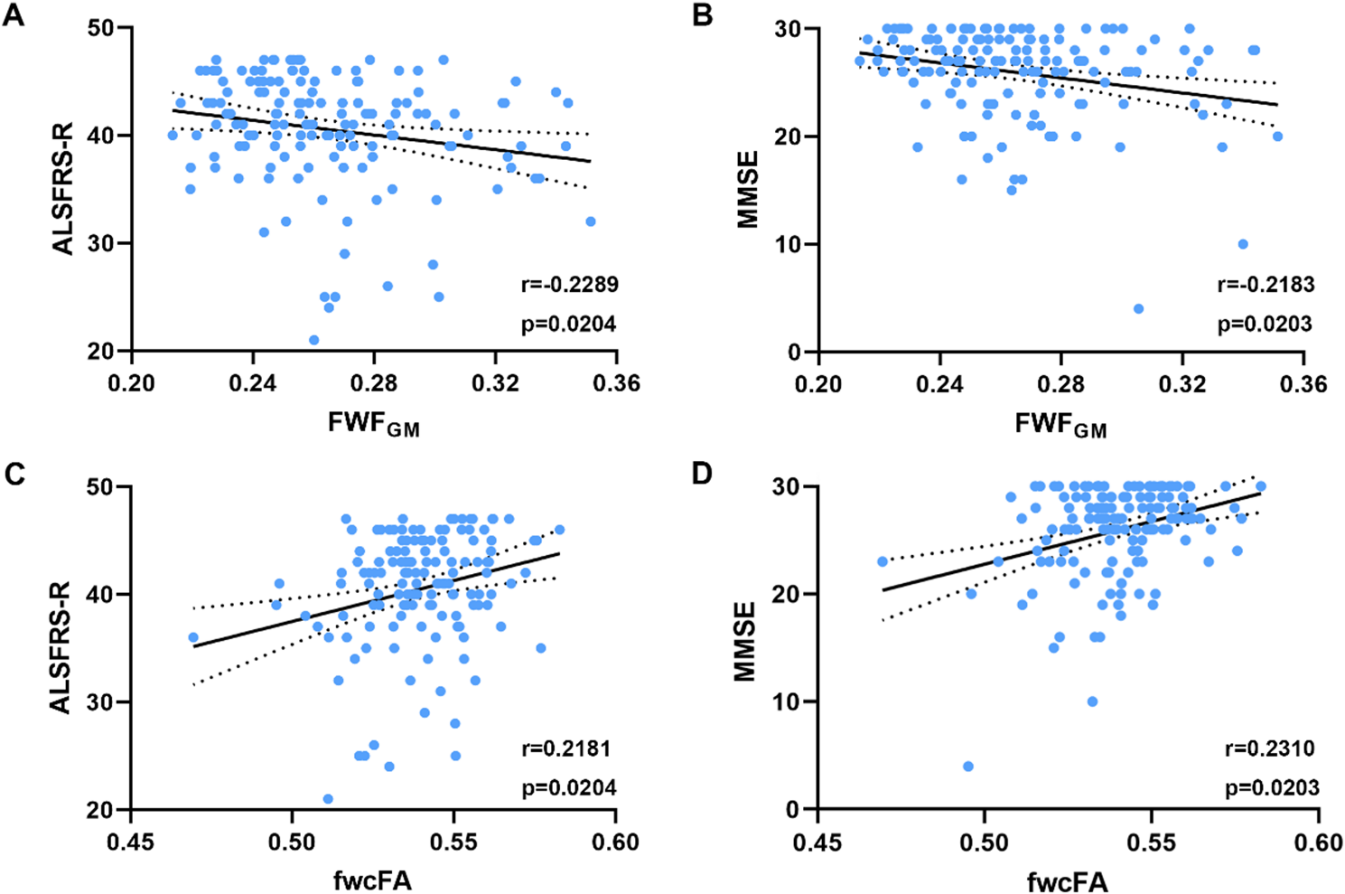
Associations between imaging and clinical data in patients with ALS. **A** Correlation between FWF_GM_ and ALSFRS-R; (**B**) Correlation between FWF_GM_ and MMSE; (**C**) Correlation between fwcFA and ALSFRS-R; (**D**) Correlation between fwcFA and MMSE. FWF, free-water fraction; fwcFA, free-water–corrected fractional anisotropy; ALSFRS-R, Amyotrophic Lateral Sclerosis Functional Rating Scale–Revised; MMSE, Mini-Mental State Examination. All reported correlations were corrected for multiple comparisons using the false discovery rate procedure

Higher cortical FWF (FWF_GM)_ was significantly associated with lower ALSFRS-R scores (*r* = -0.2289, FDR-corrected *p* = 0.0204; Fig. 2A), lower MMSE scores (*r* = -0.2183, FDR-corrected *p* = 0.0203; Fig. 2B), and more advanced King’s College stages (*r* = 0.1985, FDR-corrected *p* = 0.0393).

Lower FWF_WM_ in the left anterior thalamic radiation and cerebellar region was associated with higher PHQ-9 scores (*r* = -0.2665, FDR-corrected *p* = 0.0057) and more advanced King’s College stages (*r* = -0.1873, FDR-corrected *p* = 0.0393).

Lower fwcFA of the affected tracts was significantly linked to worse clinical and cognitive outcomes, including lower ALSFRS-R scores (*r* = 0.2181, FDR-corrected *p* = 0.0204; Fig. 2C), lower MMSE scores (*r* = 0.2311, FDR-corrected *p* = 0.0203; Fig. 2D), and more advanced King’s College stages (*r*=-0.2642, FDR-corrected *p* = 0.0065).

**Fig. 3:**
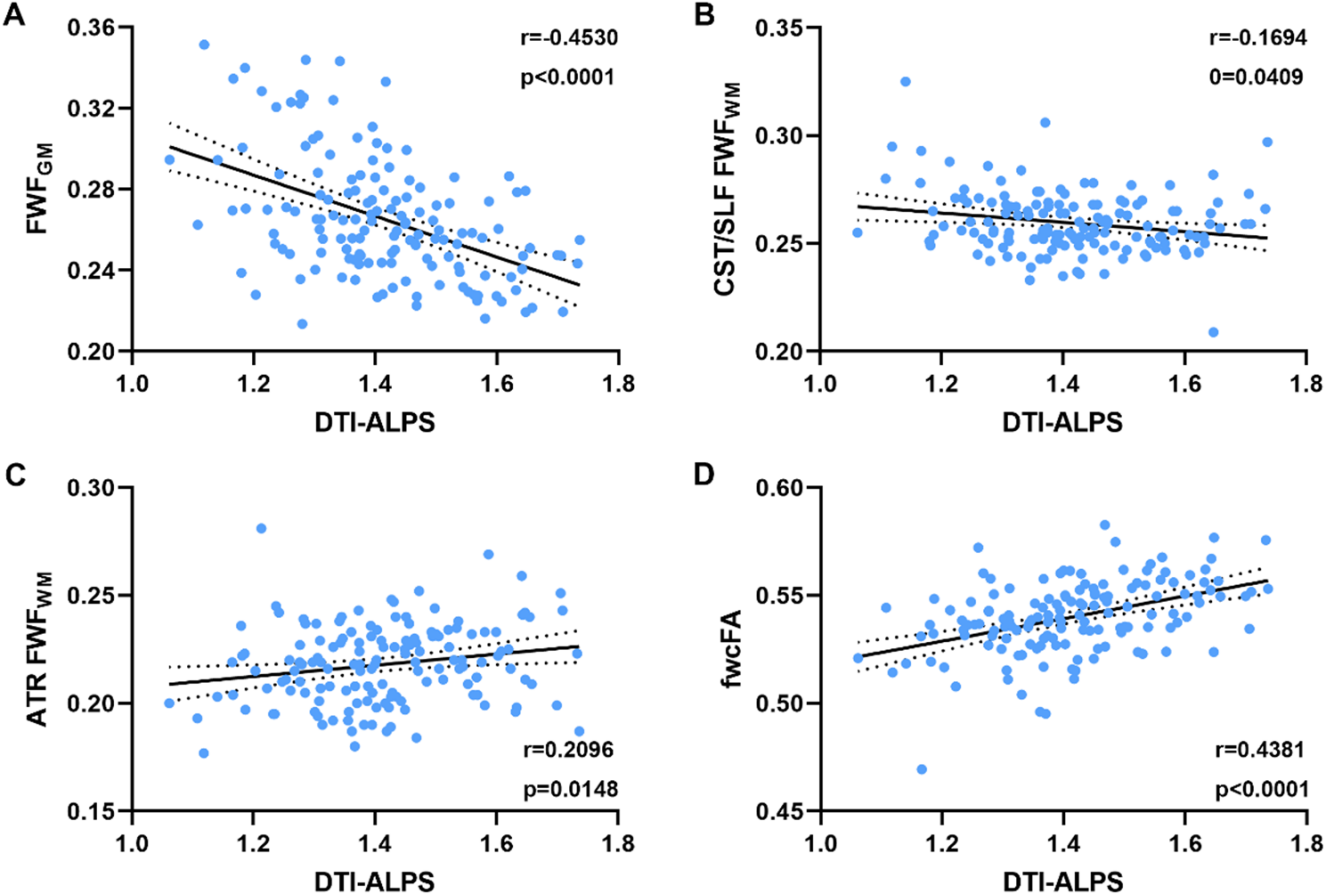
Associations of the DTI-ALPS index with FWF and fwcFA in patients with ALS. **A** Correlation between DTI-ALPS and FWF_GM_; (**B**) Correlation between DTI-ALPS and CST/SLF FWF_WM_; (**C**) Correlation between DTI-ALPS and ATR FWF_WM_; (**D**) Correlation between DTI-ALPS and fwcFA. DTI-ALPS, diffusion tensor image analysis along the perivascular space; FWF, free-water fraction; fwcFA, free-water–corrected fractional anisotropy; ATR, anterior thalamic radiation. All reported correlations were corrected for multiple comparisons using the false discovery rate procedure

A lower DTI-ALPS index was associated with higher cortical FWF (FWF_GM_) (*r* = -0.4530, FDR-corrected *p* < 0.0001; Fig. 3A) and higher FWF (FWF_WM_) in the right CST and SLF (*r* = -0.1694, FDR-corrected *p* = 0.0409; Fig. 3B), whereas it was associated with lower FWF in left anterior thalamic radiation and cerebellar region (*r* = 0.2096, FDR-corrected *p* = 0.0148; Fig. 3C), as well as with lower fwcFA in the affected tracts (*r* = 0.4381, FDR-corrected *p* < 0.0001; Fig. 3D).

### Mediation analyses

**Fig. 4:**
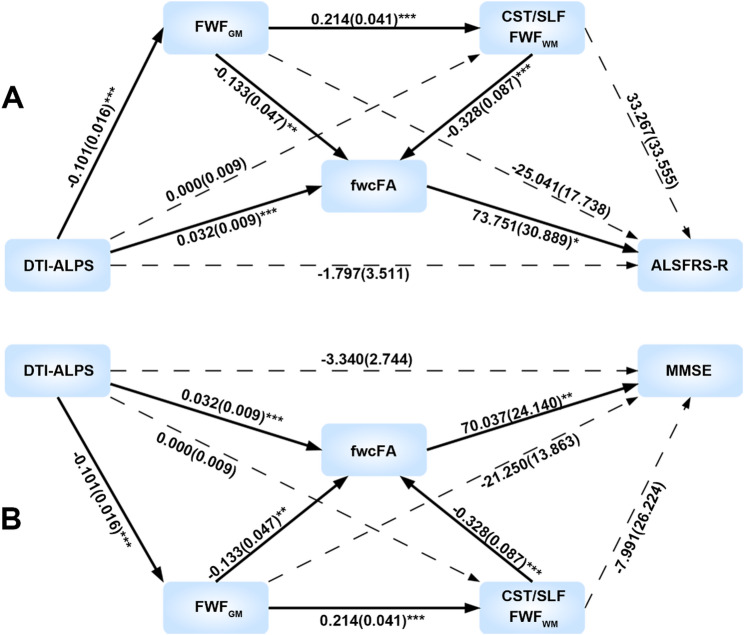
Mediation pathways linking DTI-ALPS, diffusion metrics, and clinical outcomes in ALS. **A** Serial mediation model illustrating the relationships between the DTI-ALPS index and functional disability (ALSFRS-R), with cortical FWF (FWF_GM_), white matter FWF in the CST/SLF (FWF_WM_), and fwcFA entered as mediators. **B** Serial mediation model illustrating the relationships between the DTI-ALPS index and global cognitive performance (MMSE), with cortical FWF (FWF_GM_), white matter FWF in the CST/SLF (FWF_WM_), and fwcFA entered as mediators. Solid arrows indicate statistically significant paths, whereas dashed arrows denote nonsignificant paths. Path coefficients represent unstandardized regression coefficients with standard errors in parentheses. Indirect effects were estimated using bias-corrected bootstrap procedures with 5,000 resamples. *p < 0.05; **p < 0.01; ***p < 0.001. DTI-ALPS, diffusion tensor image analysis along the perivascular space; FWF, free-water fraction; fwcFA, free-water–corrected fractional anisotropy; CST, corticospinal tract; SLF, superior longitudinal fasciculus; ALSFRS-R, Amyotrophic Lateral Sclerosis Functional Rating Scale–Revised; MMSE, Mini-Mental State Examination

We next examined whether DTI-ALPS was associated with functional and cognitive outcomes through a pathway involving cortical FWF (FWF_GM_), white matter FWF (FWF_WM_, i.e., FWF increase in white matter), and fwcFA. For ALSFRS-R, neither the total effect (β = 3.87, 95% CI [-2.13, 9.88], *p* = 0.204) nor the direct effect of DTI-ALPS (β = -1.80, 95% CI [-8.74, 5.14], *p* = 0.610) was significant. In contrast, the total indirect effect through the mediators was significant (β = 5.67, 95% CI [2.44, 9.34]), with the full serial pathway (DTI-ALPS → FWF_GM_ → FWF_WM_ → fwcFA → ALSFRS-R) showing a significant mediating effect (β = 0.52, 95% CI [0.04, 1.33]; Fig. 4A). For MMSE, the total and direct effects of DTI-ALPS were non-significant (total effect: β = 2.67, 95% CI [− 2.16, 7.50], *p* = 0.277; direct effect: β = -3.34, 95% CI [-8.77, 2.09], *p* = 0.226). Nevertheless, the total indirect effect was significant (β = 6.01, 95% CI [2.59, 10.19]), and the full serial pathway through FWF_GM_, FWF_WM_, and fwcFA also demonstrated a significant mediating effect (β = 0.50, 95% CI [0.07, 1.35]; Fig. 4B). These results indicate that DTI-ALPS is linked to functional status and cognitive performance through interrelated brain microstructural alterations, even in the absence of significant direct effects. Additional analyses adjusting for age and sex as covariates of no interest yielded similar results (Additional file 1: Fig. S4), confirming that the observed mediation effects were robust to these covariates. Parallel mediation analyses for decreased FWF in the left anterior thalamic radiation and cerebellar region did not reveal significant effects.

## Discussion

In this study, we systematically investigated putative glymphatic function and FW-related microstructural alterations in ALS and demonstrated several convergent lines of evidence linking putative glymphatic dysfunction to cortical and white matter abnormalities and to clinical impairment. Patients with ALS exhibited significantly reduced DTI-ALPS index, elevated cortical FWF, bidirectional changes in white matter FWF, and reduced fwcFA. These imaging abnormalities were associated with functional decline, disease progression, cognitive impairment, and emotional symptoms. Importantly, mediation analyses indicated that the relationship between putative glymphatic dysfunction and clinical measures may be explained by a pathway involving cortical FWF, white matter FWF, and tract-specific fwcFA, rather than a direct association. Collectively, these findings support the notion that putative glymphatic dysfunction co-occurs with microstructural disruption and clinical impairment in ALS.

The observed reduction in the DTI-ALPS index in patients with ALS provides converging in vivo evidence for putative glymphatic dysfunction and reinforces the notion that disruption of brain-CSF exchange is a core feature of ALS pathophysiology [14, 40]. As an imaging marker sensitive to water diffusion along perivascular spaces, the DTI-ALPS index has consistently been shown to be decreased in ALS across independent cohorts, including patients at early disease stages and in longitudinal follow-up [16, 28, 41], suggesting that putative glymphatic dysfunction may occur in parallel with neurodegenerative processes in ALS. In this study, the association of lower DTI-ALPS values with accelerated disease progression further highlights its relevance to disease severity. These clinical observations are biologically plausible in light of extensive experimental evidence demonstrating impaired interstitial fluid flow, delayed clearance of metabolic byproducts, and stagnation of glymphatic transport in transgenic ALS models [12, 13, 42]. Such alterations have been closely linked to astrocytic pathology, particularly disrupted aquaporin-4 localization at perivascular endfeet and aberrant astroglial activation [43, 44], both of which are critical determinants of glymphatic efficiency. In addition, CSF-mediated neuroinflammatory signaling, as observed in ALS [43], may further compromise perivascular transport and exacerbate waste accumulation. Given the established role of the glymphatic system in clearing misfolded proteins and maintaining neuronal homeostasis [45–48], putative glymphatic dysfunction as indexed by DTI-ALPS may facilitate the buildup of pathogenic proteins such as TDP-43, promote neuroinflammation, and contribute to the widespread neural dysfunction that extends beyond the motor system. Thus, reduced DTI-ALPS in ALS likely reflects a fundamental disturbance in perivascular fluid dynamics, providing a potential link between astroglial-vascular dysfunction and the clinical abnormalities characteristic of the disease.

In addition to putative glymphatic dysfunction, ALS was marked by a distinct pattern of microstructural abnormalities captured by FW-based metrics and FW-corrected diffusion indices, indicating concomitant alterations of extracellular and tissue-specific compartments. The diffuse increase in cortical FW may reflect expansion of extracellular water, which can be associated with neuroinflammatory activity, astroglial reactivity, microvascular-interstitial coupling disruptions, or atrophy-related processes [43, 44, 49, 50], rather than being specific to any single mechanism. Evidence from FW and neurite orientation dispersion and density imaging (NODDI) studies in Alzheimer’s disease [51–54], Lewy body dementia [52], and vascular cognitive impairment [55] indicates that such cortical FW elevations emerge early and track cognitive vulnerability and inflammatory burden, supporting the interpretation of FW as a sensitive marker of cortical microenvironmental disturbance. In ALS, cortical FW elevations involved not only the primary motor cortex but also frontal and parietal association regions, including the orbitofrontal cortex, superior and inferior frontal gyri, superior parietal lobule, and angular gyrus. These regions constitute key nodes of frontoparietal and default-mode networks supporting executive control, attention, and socio-emotional processing [56, 57]. The spatial distribution of cortical FW alterations provides a plausible neurobiological substrate for the cognitive impairment and emotional disturbances observed in ALS, consistent with the growing recognition of ALS as a multisystem disorder extending beyond motor circuitry.

White matter FW changes were heterogeneous, underscoring region-dependent microstructural remodeling. Increased FW within the CST and SLF may reflect extracellular fluid accumulation associated with processes such as axonal degeneration, myelin disintegration, inflammation, among others. Our findings are in line with prior diffusion and NODDI studies demonstrating reduced neurite density and increased isotropic diffusion in these pathways in ALS [58, 59]. While CST involvement represents a hallmark of ALS pathology [60], alterations in the SLF point to disruption of frontoparietal connectivity, providing a structural basis for non-motor cognitive dysfunction [61, 62]. Conversely, reduced FW in the anterior thalamic radiation and cerebellar region suggests a distinct pattern of microstructural alteration, although its biological underpinnings remain less well understood. Given that lower FW values in this tract were associated with greater depressive and anxiety symptoms, FW reduction may reflect cumulative microstructural changes within thalamocortical circuits that are involved in affective regulation. However, these interpretations should be considered preliminary, and future studies integrating FW with more specific microstructural or molecular markers are needed to clarify the underlying mechanisms.

Complementing these findings, FW-corrected FA exhibited widespread reductions across major white matter tracts, indicating substantial impairment of axonal coherence and myelin integrity independent of extracellular water contamination. fwcFA has been shown to more accurately reflect intrinsic white matter degeneration than conventional FA [20, 63–66], and its strong associations with functional decline, cognitive impairment, and clinical staging in the present study underscore its relevance to clinical expression. The observed alterations in FW and reductions in fwcFA suggest that extracellular fluid dysregulation and axonal degeneration may evolve in parallel, potentially interacting, together constituting the structural substrates underlying both motor and non-motor symptoms in ALS.

Building on these associations, the mediation analyses provide a statistical framework linking putative glymphatic dysfunction to clinical outcomes through interrelated microstructural alterations. Although DTI-ALPS did not show significant direct associations with either functional status or cognitive performance, significant indirect effects were observed through a pathway involving cortical FWF expansion, white matter FW alterations, and reductions in fwcFA. However, as these analyses are based on cross-sectional data, the observed associations should be interpreted as correlational rather than indicative of a causal pathway. Nevertheless, they highlight the clinical relevance of FW-based metrics and FW-corrected diffusion indices, which appear to capture complementary aspects of microstructural alterations linked to ALS symptomatology.

In light of these findings, the DTI-ALPS index and FW-based measures may serve as candidate imaging markers of fluid dysregulation related to tissue microstructure and clinical status. Their relationships with functional status, cognition, and disease stage, though modest in magnitude (*r* ≈ 0.2–0.3), suggest potential utility for disease monitoring, patient stratification, and outcome assessment in clinical trials. Given the relatively large sample size, modest effect sizes are expected, and these associations should therefore be interpreted cautiously. Nevertheless, such relationships indicate that these metrics capture clinically relevant aspects of fluid dysregulation. Although their sensitivity and discriminative performance as biomarkers remain to be established, they may have translational value for monitoring disease processes, informing biomarker development, and evaluating interventions targeting astroglial, vascular, or sleep-related mechanisms.

Several limitations of the present study should be acknowledged. First, the interpretation of DTI-ALPS as a direct measure of glymphatic function warrants caution. While DTI-ALPS reflects diffusivity along perivascular spaces in deep periventricular white matter, it may also be influenced by the microstructural properties of nearby projection and commissural fiber pathways (e.g., corticospinal tracts and corpus callosum) [67]. Prior validation studies have yielded inconsistent results regarding its correspondence with more direct measures of glymphatic function, such as contrast-enhanced imaging of subcortical perivascular spaces [68, 69]. Therefore, DTI-ALPS should be considered an indirect proxy rather than a definitive marker of glymphatic activity, and reductions in DTI-ALPS should be interpreted as consistent with—but not specific to—glymphatic dysfunction. Second, the cross-sectional design precludes inferences regarding causal or temporal relationships among putative glymphatic dysfunction, microstructural alterations, and clinical outcomes. Although the serial mediation analyses provide a structured statistical framework, longitudinal studies are needed to confirm the directionality of these associations and to determine whether putative glymphatic dysfunction precedes or follows microstructural degeneration. Third, the use of atlas-based ROIs derived from the JHU-ICBM-DTI-81 white-matter atlas may have limitations in capturing subject-specific tract anatomy. Although atlas-based ROIs provide reproducible and operator-independent localization, residual anatomical variability and atrophy in ALS patients may result in imperfect alignment or mislocalization of the DTI-ALPS ROIs. To mitigate these issues, we conducted careful quality control, including visual inspection of spatial normalization, to ensure accurate ROI placement. Fourth, the voxel-wise white matter microstructural analyses were restricted to the TBSS-derived skeleton. While TBSS is widely used and provides robust voxel-wise comparisons across subjects, focusing on the skeleton may not fully capture microstructural alterations in the peripheral or deeper portions of white matter tracts. Consequently, subtle tract-specific changes along the full trajectory of fibers could be overlooked. Future studies employing tractography-based or fixel-based analyses could complement these findings by providing more anatomically precise profiles throughout the entire tract volume. Fifth, while the sample size is relatively large for an ALS neuroimaging study, the cohort may not fully capture the heterogeneity of ALS, including atypical presentations or different disease subtypes, and demographic matching was limited to age and sex. Sixth, cognitive and emotional assessments were relatively coarse; for example, MMSE, PHQ-9, and GAD-7 provide global measures but may not detect subtle or domain-specific impairments that could relate to microstructural changes. Seventh, although DTI-ALPS and FW-corrected diffusion metrics are sensitive markers of putative glymphatic dysfunction and tissue integrity, their values may be influenced by confounding factors such as head motion, or partial volume effects. Finally, despite statistically significant group differences in multiple imaging and clinical measures, effect sizes for some correlations were modest, highlighting the need for replication in independent cohorts and careful interpretation when extrapolating to individual prognosis.

## Conclusions

In conclusion, this study demonstrates that putative glymphatic dysfunction, as reflected by reduced DTI-ALPS, co-occurs with widespread microstructural alterations in both cortical and white matter regions, which are associated with functional decline, cognitive impairment, and more advanced disease stage in ALS. These findings provide in vivo evidence supporting the relevance of perivascular fluid dynamics in ALS and underscore the value of multi-compartment diffusion imaging for capturing microstructural variations relevant to clinical outcomes.

## Supplementary Information

Below is the link to the electronic supplementary material.

Supplementary Material 1: Figure S1–Figure S4

## Data Availability

The data supporting the findings of this study are available from the corresponding author upon reasonable request.

## Abbreviations

ALS: Amyotrophic lateral sclerosis
ALSFRS-R: Amyotrophic Lateral Sclerosis Functional Rating Scale-Revised
CIs: Confidence intervals
CSF: Cerebrospinal fluid
CST: Corticospinal tract
DTI-ALPS: Diffusion tensor imaging along perivascular spaces
FA: Fractional anisotropy
FOV: Field of view
FW: Free water
fwcFA: free-water corrected fractional anisotropy
FWF: Free water fraction
GAD-7: Generalized Anxiety Disorder 7-item
HCs: Healthy controls
MMSE: Mini-Mental State Examination
NODDI: Neurite orientation dispersion and density imaging
PHQ-9: Patient Health Questionnaire-9
ROIs: Regions of interest
SCR: Superior corona radiata
SLF: Superior longitudinal fasciculus
TBSS: Tract-Based Spatial Statistics
TE: Echo time
TFCE: Threshold-free cluster enhancement
TI: Inversion time
TR: Repetition time
