# Supplementary material for: Putative glymphatic dysfunction links extracellular fluid dysregulation to white matter degeneration and clinical impairment in amyotrophic lateral sclerosis
Source: BMC Med. 2026 May 27;24:405. doi: 10.1186/s12916-026-04948-z (PMC13397807; doi:10.1186/s12916-026-04948-z)
Supplement: Supplementary file 1 — Supplementary Material 1: Figure S1–Figure S4 [file 12916_2026_4948_MOESM1_ESM.docx]

**
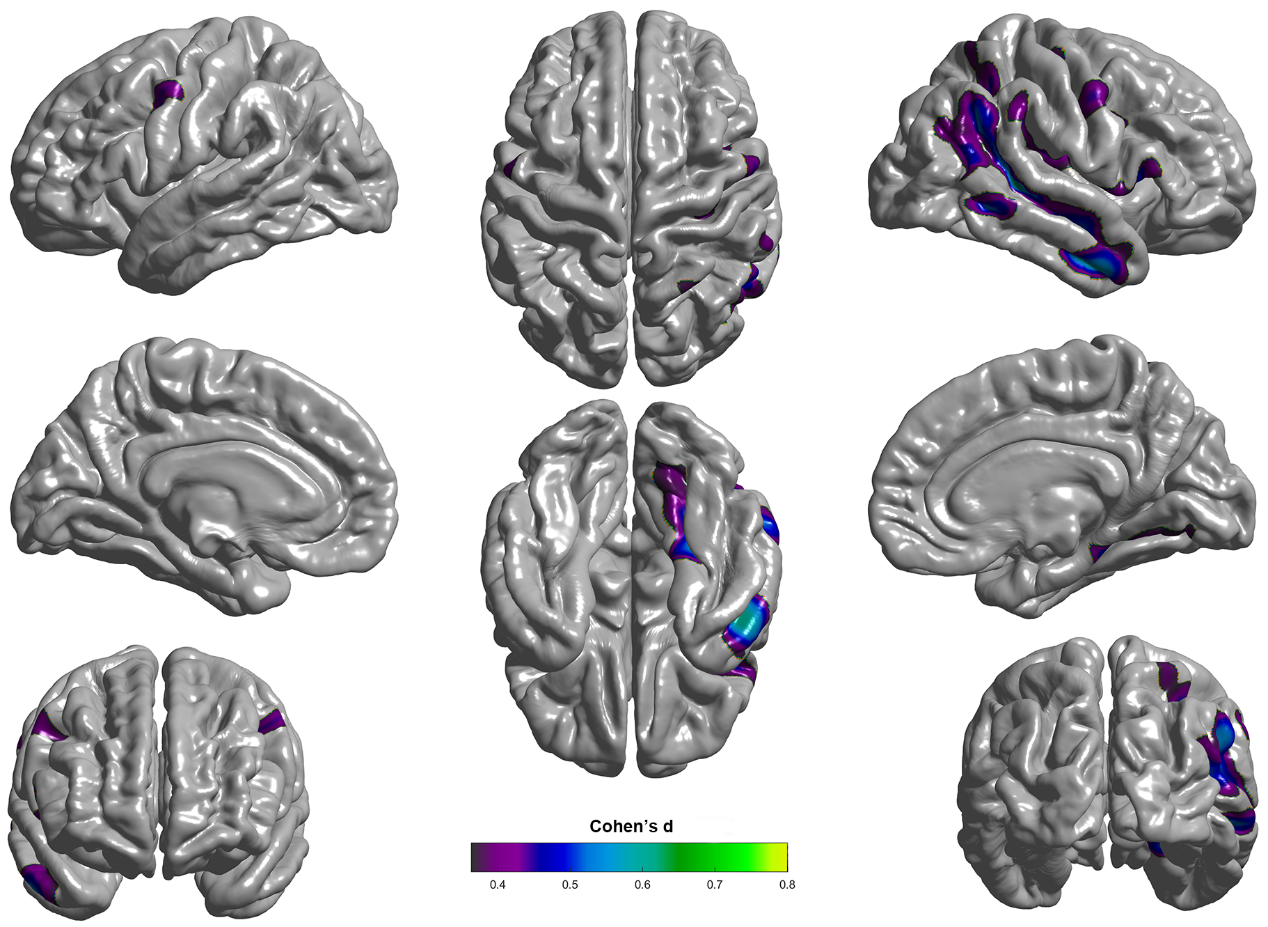
**

**Figure S1. Effect size map of cortical free water fraction (FWF).** Significant clusters for the contrast between patients with amyotrophic lateral sclerosis (ALS) and healthy controls (HCs) are displayed on the cortical surface. The color bar denotes Cohen’s d values for the contrast (ALS>HCs).


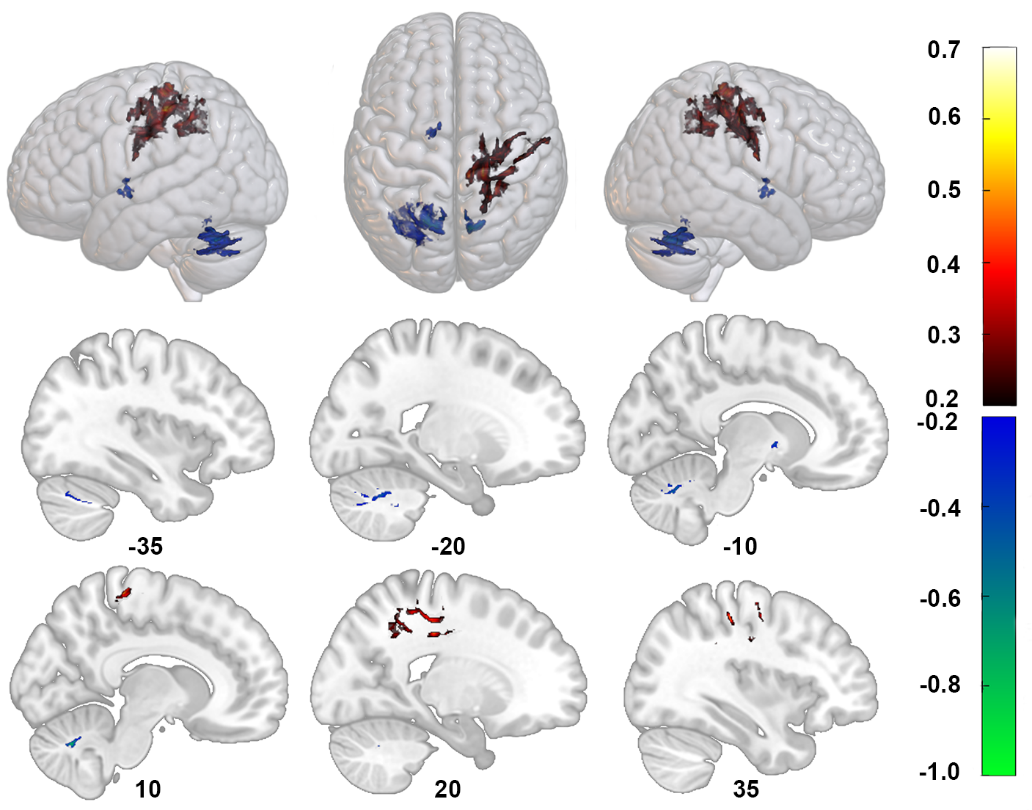


**Figure S2. Effect size map of white matter free water fraction (FWF).** Warm colors indicate regions with higher FWF in ALS patients compared to healthy controls (HCs), whereas cool colors indicate regions with lower FWF in ALS. The color bars represent Cohen’s d values for the contrasts ALS > HCs (positive) and ALS < HCs (negative), respectively.


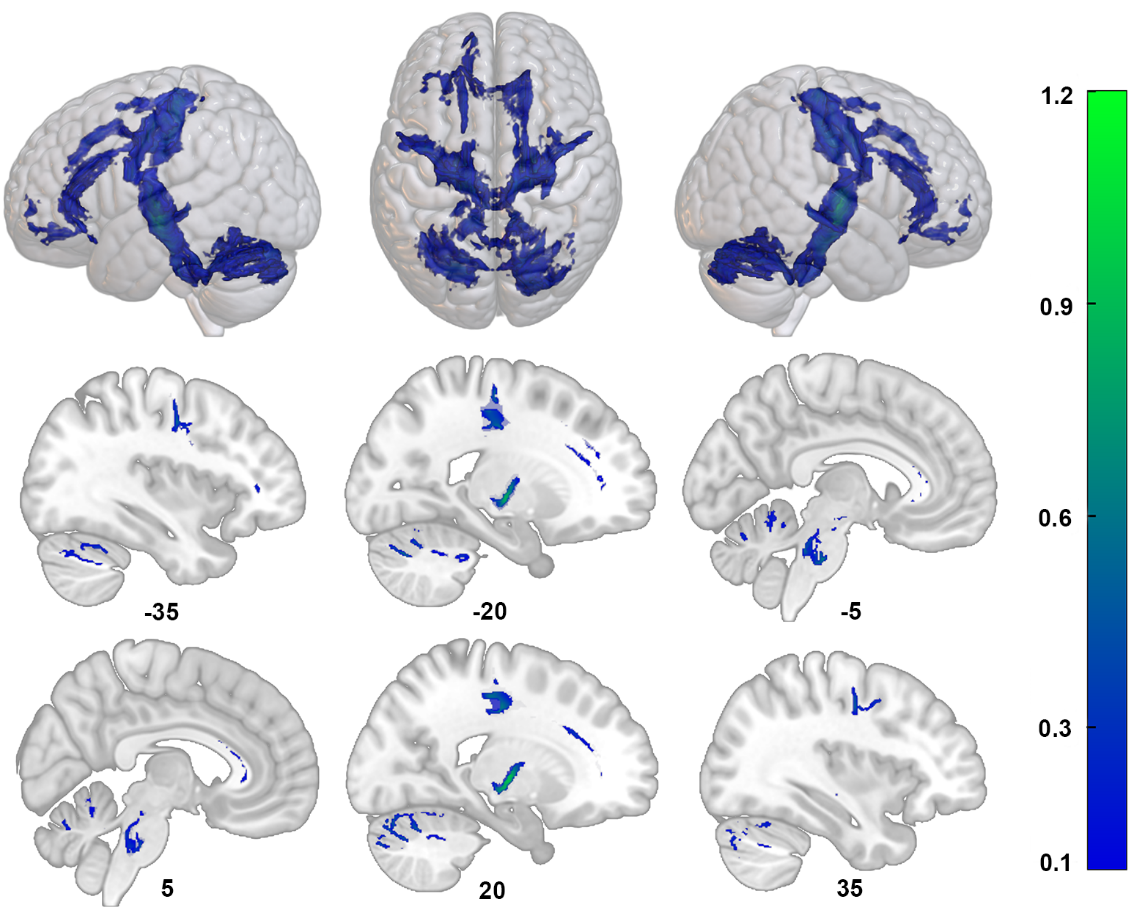


**Figure S3. Effect size map of white matter free water-corrected fractional anisotropy (fwcFA).** The color bar represents Cohen’s d values for the contrast ALS < HCs.


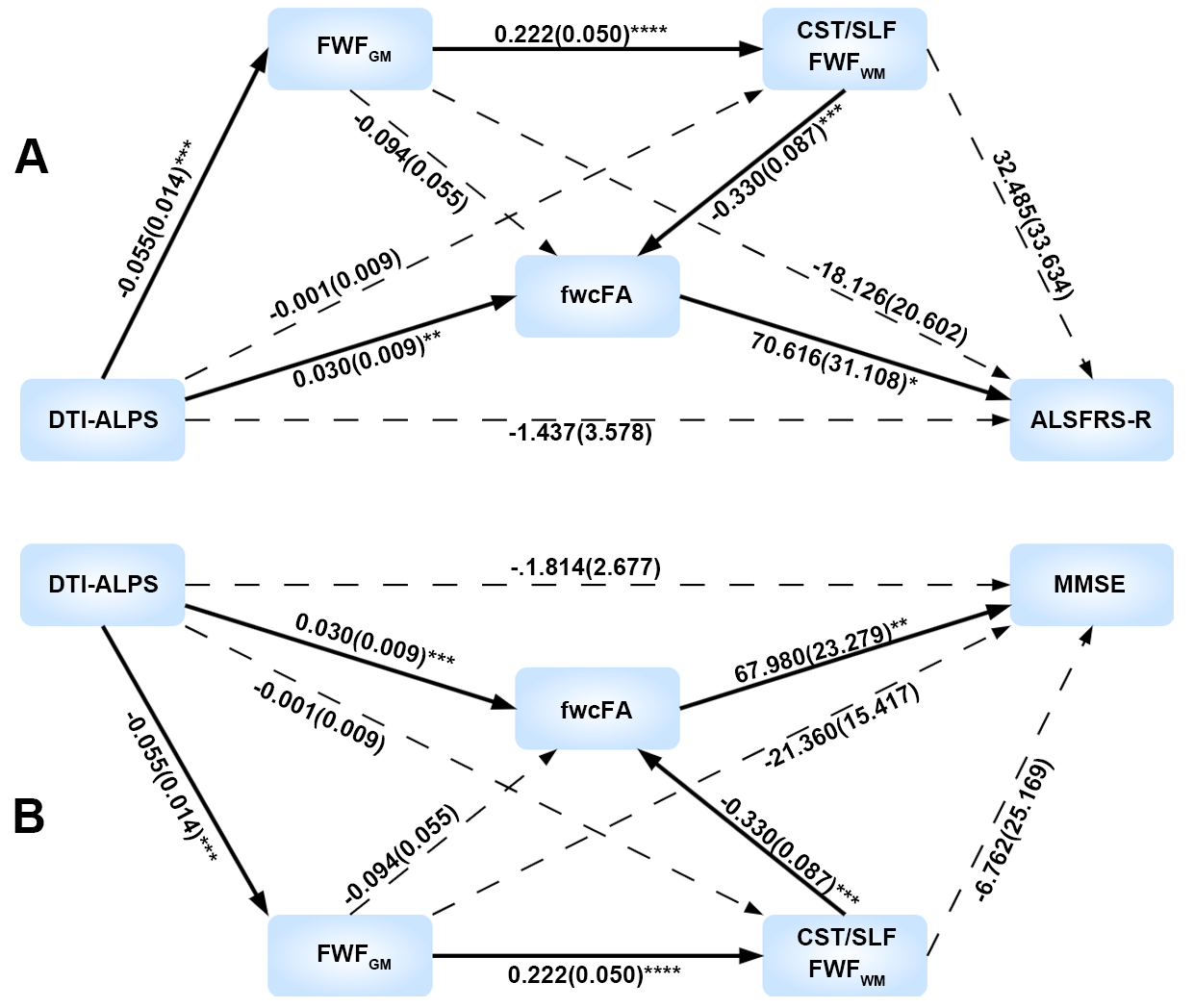


**Figure S4. Mediation pathways linking DTI-ALPS, diffusion metrics, and clinical outcomes in ALS, controlling for age and sex.** (**A**) Serial mediation model illustrating the relationships between the DTI-ALPS index and functional disability (ALSFRS-R), with cortical FWF (FWF_GM_), white matter FWF in the CST/SLF (FWF_WM_), and fwcFA entered as mediators. (**B**) Serial mediation model illustrating the relationships between the DTI-ALPS index and global cognitive performance (MMSE), with cortical FWF (FWF_GM_), white matter FWF in the CST/SLF (FWF_WM_), and fwcFA entered as mediators. Solid arrows indicate statistically significant paths, whereas dashed arrows denote nonsignificant paths. Path coefficients represent unstandardized regression coefficients with standard errors in parentheses. Indirect effects were estimated using bias-corrected bootstrap procedures with 5,000 resamples. **p* < 0.05; ***p* < 0.01; ****p* < 0.001. DTI-ALPS, diffusion tensor image analysis along the perivascular space; FWF, free-water fraction; fwcFA, free-water–corrected fractional anisotropy; CST, corticospinal tract; SLF, superior longitudinal fasciculus; ALSFRS-R, Amyotrophic Lateral Sclerosis Functional Rating Scale–Revised; MMSE, Mini-Mental State Examination.
